# Effects of new beta-type Ti-40Nb implant materials, brain-derived neurotrophic factor, acetylcholine and nicotine on human mesenchymal stem cells of osteoporotic and non osteoporotic donors

**DOI:** 10.1371/journal.pone.0193468

**Published:** 2018-02-28

**Authors:** Vivien Kauschke, Annett Gebert, Mariana Calin, Jürgen Eckert, Sebastian Scheich, Christian Heiss, Katrin Susanne Lips

**Affiliations:** 1 Experimental Trauma Surgery, Justus-Liebig-University Giessen, Giessen, Germany; 2 Leibniz Institute for Solid State and Materials Research Dresden, Dresden, Germany; 3 Erich Schmid Institute of Materials Science, Austrian Academy of Sciences, Leoben, Austria; 4 Department Materials Physics, Montanuniversität Leoben, Leoben, Austria; 5 Department of Trauma Hand and Reconstructive Surgery, University Hospital of Giessen-Marburg, Campus: Giessen, Giessen, Germany; Medical University of South Carolina, UNITED STATES

## Abstract

**Introduction:**

Treatment of osteoporotic fractures is still challenging and an urgent need exists for new materials, better adapted to osteoporotic bone by adjusted Young’s modulus, appropriate surface modification and pharmaceuticals.

**Materials and methods:**

Titanium-40-niobium alloys, mechanically ground or additionally etched and titanium-6-aluminium-4-vanadium were analyzed in combination with brain-derived neurotrophic factor, acetylcholine and nicotine to determine their effects on human mesenchymal stem cells *in vitro* over 21 days using lactate dehydrogenase and alkaline phosphatase assays, live cell imaging and immunofluorescence microscopy.

**Results:**

Cell number of human mesenchymal stem cells of osteoporotic donors was increased after 14 d in presence of ground titanium-40-niobium or titanium-6-aluminium-4-vanadium, together with brain-derived neurotrophic factor. Cell number of human mesenchymal stem cells of non osteoporotic donors increased after 21 d in presence of titanium-6-aluminium-4-vanadium without pharmaceuticals. No significant increase was measured for ground or etched titanium-40-niobium after 21 d. Osteoblast differentiation of osteoporotic donors was significantly higher than in non osteoporotic donors after 21 d in presence of etched, ground titanium-40-niobium or titanium-6-aluminium-4-vanadium accompanied by all pharmaceuticals tested. In presence of all alloys tested brain-derived neurotrophic factor, acetylcholine and nicotine increased differentiation of cells of osteoporotic donors and accelerated it in non osteoporotic donors.

**Conclusion:**

We conclude that ground titanium-40-niobium and brain-derived neurotrophic factor might be most suitable for subsequent *in vivo* testing.

## 1. Introduction

Osteoporosis is characterized by low mineral density and altered microarchitecture that causes fragile bone and often results in fractures [1]. Surgical treatment of osteoporotic fractures is still challenging for clinicians since implant fixation in osteoporotic bone often fails [2, 3]. Therefore, new materials are needed, which are adapted to the characteristics of osteoporotic bone and ideally stimulate fracture healing. In particular, implant materials for orthopedic applications should be robust and biocompatible [4]. With regard to non degradable implants, titanium-based materials are predominantly used [4, 5]. Titanium and titanium alloys are preferred as implant materials because of their biocompatibility and high corrosion resistance [4, 6, 7]. In particular, beta titanium alloys are favored for the treatment of fractures due to their low elastic modulus, which comes close to that of bone [4, 8, 9] and is much lower than for currently applied titanium or titanium alloys like titanium-6-aluminium-4-vanadium (Ti-6Al-4V) [10]. Especially, a low Young’s modulus is preferable since high Young’s moduli, as measured for the momentarily applied alloy titanium Ti-6Al-4V in orthopedic surgery [11] were shown to result in atrophy of bone and deficient remodeling [6, 10]. This can lead to implant failure [11]. The beta-alloy titanium-40-niobium (Ti-40Nb), in contrast, possesses an elastic modulus of 60–62 GPa, which can be further reduced by microalloying and thermomechanical treatment [5] reaching an elastic modulus closer to the one of bone.

Surface modifications of implant materials play an important role as well. They can prevent corrosion and offer higher biocompatibility by inhibiting an inflammatory immune response [4, 12]. Moreover, surface modification has effects on cell growth and morphology [5].

Human mesenchymal stem cells (hMSCs) are ideal for testing since they proliferate quickly and they are able to differentiate into bone forming osteoblasts [13–15]. These characteristics make hMSCs a potential therapeutic in bone regeneration [16, 17]. However, in osteoporosis osteogenic differentiation of hMSCs is impaired in favor of adipogenic differentiation [18]. Therefore, substances are required that stimulate hMSCs differentiation into osteoblasts. As such, bone morphogenic protein 2 (BMP2) is already applied in the clinic. However, its effects on osteogenic differentiation of hMSCs from osteoporotic patients *in vivo* are rather low [19]. Thus, there is a need for new factors that stimulate osteogenic differentiation in osteoporosis.

Brain-derived neurotrophic factor (BDNF) was shown to stimulate secretion of vascular endothelial growth factor (VEGF) from osteoblasts during fracture healing [20]. This is important since fractures do not heal properly without angiogenesis [21, 22]. Moreover, BDNF plays a potential role during bone remodeling and bone formation. It is involved in differentiation processes and was detected in osteoblast-like cells or osteoblasts in different healing models [23–27].

Several studies demonstrated that acetylcholine (ACh) is involved in the regulation of proliferation and differentiation of osteoblasts [28–30]. Sato et al. (2010) showed that ACh supports cell cycle progression in osteoblasts, but inhibits alkaline phosphatase (ALP) activity during osteoblast differentiation [29].

Effects of nicotine (Nic) on bone metabolism are discussed controversially. It was shown that nicotine concentrations, as found in heavy smokers, inhibited osteoblast differentiation, worsened fracture healing [31] and increased osteoclast differentiation *in vitro* [32]. Kim et al. (2012) demonstrated bimodal effects of Nic at low concentrations by means of increased osteoblast proliferation and decreased differentiation [33]. However, Rothem et al. (2009) indicated dose-dependent effects of Nic. Nic concentrations as present in light or moderate smokers increased osteoblast proliferation but at higher concentrations, as seen in case of heavy smokers, it caused adverse effects [34], which was confirmed by Shen et al. (2013) [31].

These findings indicate that BDNF, ACh and Nic might be potential pharmaceuticals for the treatment of osteoporotic fractures, which was the underlying reason to analyze these factors in the present study *in vitro*.

We therefore analyzed whether new Ti-40Nb alloys together with BDNF, ACh or Nic are potential drugs to increase cell number and to stimulate osteogenic differentiation of hMSCs, especially in osteoporotic patients.

## 2. Materials and methods

### 2.1 Human mesenchymal stem cells

Harvesting of hMSCs was approved by a written statement of the local ethics commission of the department of medicine at the Justus-Liebig-University of Giessen (74/09). Patients gave written consent for their participation in the underlying study.

hMSCs were isolated from reaming debris as described by Wenisch et al. (2005) [35] and were obtained from different female osteoporotic as well as male and female non-osteoporotic donors (n = 4 each) who underwent surgery in the department of trauma surgery at the University of Giessen. Age of patients ranged from 25 to 80 years.

In brief, reaming debris was incubated in Petri dishes (Becton-Dickson Falcon Franklin Lakes, New Jersey, USA) with F-12K medium (Gibco, Life Technologies, Carlsbad, USA) containing 20% fetal calf serum (PanSera ES, Pan Biotech, Aidenbach, Germany), 1% of 100 U/ml penicillin and 100 μg/g streptomycin (Gibco) at 37°C under 6% CO_2_ atmosphere. After approximately 1 week cells migrated out of the debris. When cell growth reached confluence cells were detached by applying 0.05% Trypsin (Gibco). Subsequently, hMSCs were transferred into cryo tubes (Greiner bio-one, Frickenhausen, Germany) containing 0.9 ml fetal calf serum (FCS) as well as 0.1 ml DMSO and stored over night at -80°C before being placed in liquid nitrogen.

Identification of hMSCs was achieved using a Fluorescence Activated Cell Sorting (FACS) machine, FACS Canto II (BD Biosciences, Franklin Lakes, New Jersey, USA) after labeling cells with mouse-anti-human CD105-APC and mouse-anti-human CD73-PB antibodies (BioLegend, San Diego, California, USA).

Before starting the experimental procedure, hMSCs were placed in 200 ml cell culture flasks (Greiner bio-one) containing MesenPro RS Medium (Gibco) including 10% FCS (PanSera ES), 1% Glutamax (Gibco) and 1% of 100 U/ml penicillin and 100 μg/g streptomycin (Gibco) at 37°C under 6% CO_2_ atmosphere. Cells were split once when confluence was reached.

Experiments were performed in 24-well-plates (Becton-Dickson Falcon). Therefore, 4x10^4^ hMSCs were seeded into each well. Cells were cultivated in F-12K medium (Gibco) containing 20% FCS for cell number analysis.

For differentiation assays osteogenic medium composed of low glucose Dulbeccos modified Eagles medium (Gibco), including 10% FCS (Biochrom, Berlin, Germany), 1% 100 U/ml penicillin and 100 μg/g streptomycin (Gibco), 10^−7^ M dexamethasone (Sigma, St. Louis, Missouri, USA), 5x10^-5^ M (+) sodium-L-ascorbat (Sigma), 10^−2^ M ß-glycero phosphate hydrate (Sigma), 5x10^-8^ M vitamin D_3_ (Sigma-Aldrich) and 1.5x10^-3^ M calcium chloride (PromoCell, Heidelberg, Germany) was used.

hMSCs were either incubated with etched Ti-40Nb, ground Ti-40Nb or Ti-6Al-4V. Pharmaceuticals were added at the following concentrations: 40 ng/ml BDNF (Sigma), 10^−4^ M ACh (Sigma) or 10^−6^ M Nic (Sigma). Decisions for selection of concentrations used were made after testing different concentrations of these pharmaceuticals on hMSCs *in vitro* (data shown in supporting information S1 Fig). The pharmaceutical concentration coming the closest to or above the ALP concentration of cells that were incubated without pharmaceuticals (control) was chosen. Testing for the appropriate ACh concentration revealed that 10^−3^ M caused the highest ALP concentration. However, live cell images depicted holes within the cell layer so that 10^−4^ M was applied for experiments.

In order to determine effects of the different Ti alloys and pharmaceuticals used hMSCs that were incubated with or without Ti alloys in the absence of pharmaceuticals served as controls.

### 2.2 Titanium-40-niobium

Ti-40Nb samples were produced as described by Helth et al. (2014) [36]. In brief, high purity Ti and Nb were arc-melted to alloy ingots under argon atmosphere and subsequently cast into rod-shape with 10 mm diameter using cold crucible casting. The rods were homogenized by annealing for 24 h at 1000°C in an argon filled quartz tube. Subsequently, rods were cut in 2–3 mm thick disks and then, either mechanically ground or additionally chemically etched. Grinding was performed with P1200 silicon carbide emery paper. For additional etching of the Ti-40Nb surface, samples were treated with so-called piranha solution composed of 98% H_2_SO_4_ + 30% H_2_O_2_ (1+1 dilution) [5].

### 2.3 Live cell imaging

Cells were regularly monitored using an inverse light microscope (Zeiss, Oberkochen Germany) and pictures taken at time points 0 days (d), 1 d, 7 d, 14 d and 21 d with the microscope accompanying Stingray F-145 camera (Allied vision technologies GmbH, Stadtroda, Germany).

### 2.4 Immunofluorescence imaging of non osteogenic and osteogenic differentiated hMSCs

For immunofluorescence imaging cell medium was removed and cells carefully washed with cold phosphate buffered saline (PBS). Subsequently, cells were fixed in 4% paraformaldehyde (PFA) for 10 min before washed again 3x with cold PBS. For permeabilization of cells 0.1% triton-X 100 (Sigma) was added for 5 min. After washing 3x with cold PBS cells were incubated for 40 min with 1% tetramethylrhodamine B isothiocyanate (TRITC) coupled phalloidin antibody (Sigma) in the dark. For the detection of nuclei cells were incubated for 15 min with 1% Hoechst 33258 antibody (Sigma) after washing cells 6x with cold PBS. Finally, cells were covered in ProLong Gold antifade reagent (Life technologies) before microscopic evaluation.

### 2.5 Determination of hMSCs numbers

For the determination of total hMSCs numbers a CytoTox 96^®^ Non-Radioactive Cytotoxicity Assay (Promega, Madison, USA) was performed. Besides measuring cytotoxic effects, this assay can be applied for total cell number analysis. According to manufacturer’s instructions cell numbers can be analyzed if cells are lyzed using a solution to release lactate dehydrogenase (LDH), which is present inside the cytoplasm of intact cells.

Before applying the CytoTox 96^®^ Non-Radioactive Cytotoxicity Assay, hMSCs were washed twice with PBS to remove medium and potential dead cells. PBS was then completely removed and cells immediately stored at -80°C to cause cell burst followed by LDH release. Subsequently, cell membrane of hMSCs was additionally disrupted by applying 1 ml of 1% triton-X 100 (Sigma) to each well of the 24-well-plate for 50 min under shaking conditions. Lysates were then removed from the wells and transferred into Eppendorf tubes for centrifugation at 1800 rpm for 5 min. After centrifugation, 50 μl of the supernatant were filled into wells of a 96-well-plate (Greiner bio-one) in triplicates followed by 50 μl of reconstituted substrate (Promega). The 96-well-plate was then shaken for 1 min and incubated in the dark for 30 min. Finally, stop solution was added and absorption measured at 490 nm using the Synergy HT plate reader (BioTek Instruments Inc., Winooski, USA). For hMSCs of each donor a calibration curve was conducted by seeding 5x10^3^, 1x10^4^, 2x10^4^, 4x10^4^, 6x10^4^, 8x10^4^, 1x10^5^ 1,2x10^5^, 1,4x10^5^ and 1,6x10^5^ cells per well. Cell numbers are directly proportional to absorbance values measured and traced back to amounts of the calibration curve.

### 2.6 Determination of osteoblast numbers

A PicroGreen assay (Invitrogen, Eugene, Oregon, USA) was performed to determine cell numbers based on DNA content. Before conducting the assay, cells were washed twice with PBS, which was then completely removed and cells immediately stored at -80°C. Lysis was achieved by incubating cells in 250 μl of triton-X 100 (Sigma) for 10 min on a rocking platform. Lysates were then centrifuged for 10 min at 3000 g and 4°C. Two hundred μl of PicoGreen working solution were pipetted into a black 96-well-plate before 5 μl of the supernatants were added in triplicates. Fluorescence intensity was measured at 528 nm after sample excitation at 485 nm using the Synergy HT plate reader (BioTek).

### 2.7 Cell differentiation analysis

For the analysis of hMSCs differentiation into osteoblasts, the activity of alkaline phosphatase (ALP) was measured using the SensoLyte pNPP Alkaline Phosphatase Assay Kit (AnaSpec, Fremont, USA). After cell lyses and centrifugation as described above (2.6), 10 μl of the supernatants were added in triplicates into 96-well-plates. Before, wells were equipped with 40 μl of dilution buffer. Subsequently, 50 μl of para-nitrophenylphosphate substrate were added. After incubation for 45 min at 37°C, enzyme activity was measured at 405 nm using the Synergy HT plate reader (BioTek). ALP activity was referenced to cell numbers obtained from the PicoGreen assay mentioned in section 2.6.

### 2.8 Statistical analysis

Statistical analysis was carried out using the statistics program SPSS (version 22.0; SPSS Institute Inc, Chicago, USA), which was also conducted to generate graphs in figures 8 and 9 (supporting information S2 and S3). Results were evaluated by Kolmogorov-Smirnov-test to assess normality. Results were not normally distributed, so that Kruskal-Wallis-, Mann-Whitney-U-tests or Friedman-tests were conducted. For the comparison of osteoporotic and non osteoporotic donors Kruskal-Wallis- and Mann-Whitney-U-tests were conducted. The Friedman-test was applied for the comparison of different Ti alloys and the comparison of pharmaceuticals. A value of p ≤ 0.05 was considered to be significant.

## 3. Results

### 3.1 Live cell imaging

**Fig 1:**
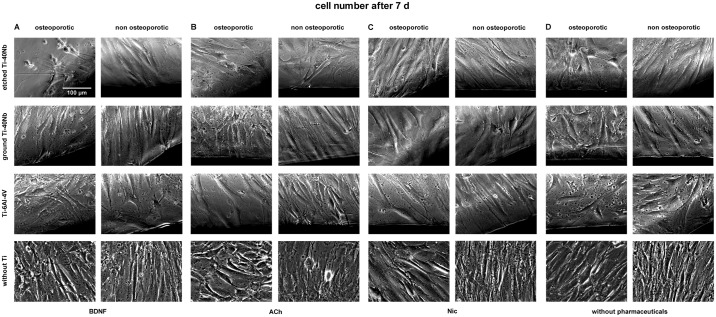
Live cell images of hMSCs number after 7 days *in vitro*. Shown are hMSCs of osteoporotic (left) and non osteoporotic (right) donors in presence of etched (1^st^ row) or ground Ti-40Nb (2^nd^ row), Ti-6Al-4V (3^rd^ row) or without Ti (4^th^ row) in presence of BDNF (A), ACh (B), Nic (C) or without pharmaceuticals serving as controls (D). The images show cells of different donors as typical representative of 4 independent experiments. Black regions at the margin of pictures show Ti alloys. Scale bar shown in A applies to all photographs in this figure.

**Fig 2:**
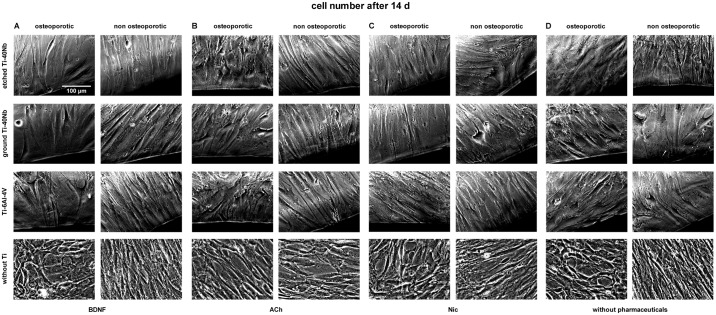
Live cell images of hMSCs number after 14 days *in vitro*. Shown are hMSCs of osteoporotic (left) and non osteoporotic (right) donors in presence of etched (1^st^ row) or ground Ti-40Nb (2^nd^ row), Ti-6Al-4V (3^rd^ row) or without Ti (4^th^ row) in presence of BDNF (A), ACh (B), Nic (C) or without pharmaceuticals serving as controls (D). The images show cells of different donors as typical representative of 4 independent experiments. Black regions at the margin of pictures show Ti alloys. Scale bar shown in A applies to all photographs in this figure.

**Fig 3:**
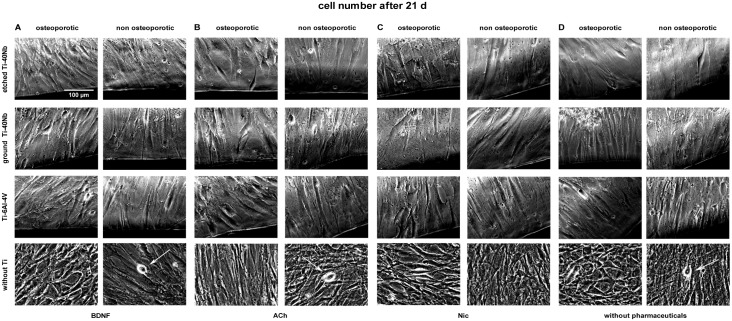
Live cell images of hMSCs number after 21 days *in vitro*. Shown are hMSCs of osteoporotic (left) and non osteoporotic (right) donors in presence of etched (1^st^ row) or ground Ti-40Nb (2^nd^ row), Ti-6Al-4V (3^rd^ row) or without Ti (4^th^ row) in presence of BDNF (A), ACh (B), Nic (C) or without pharmaceuticals serving as controls (D). White arrows indicate RS cells. The images show cells of different donors as typical representative of 4 independent experiments. Black regions at the margin of pictures show Ti alloys. Scale bar shown in A applies to all photographs in this figure.

**Fig 4:**
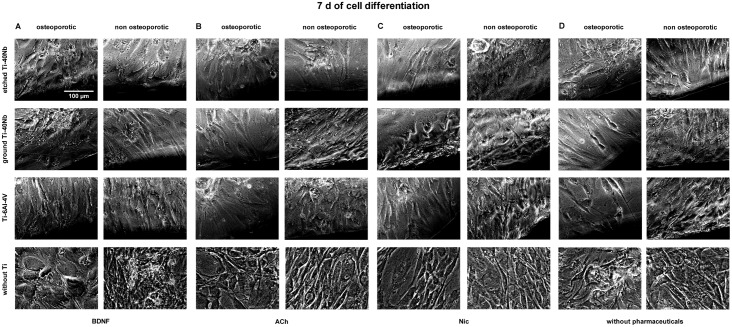
Live cell images of hMSCs after 7 days of differentiation in osteogenic medium *in vitro*. Shown are hMSCs of osteoporotic (left) and non osteoporotic (right) donors in presence of etched (1^st^ row) or ground Ti-40Nb (2^nd^ row), Ti-6Al-4V (3^rd^ row) or without Ti (4^th^ row) in presence of BDNF (A), ACh (B), Nic (C) or without pharmaceuticals serving as controls (D). The images show cells of different donors as typical representative of 4 independent experiments. Black regions at the margin of pictures show Ti alloys. Scale bar shown in A applies to all photographs in this figure.

**Fig 5:**
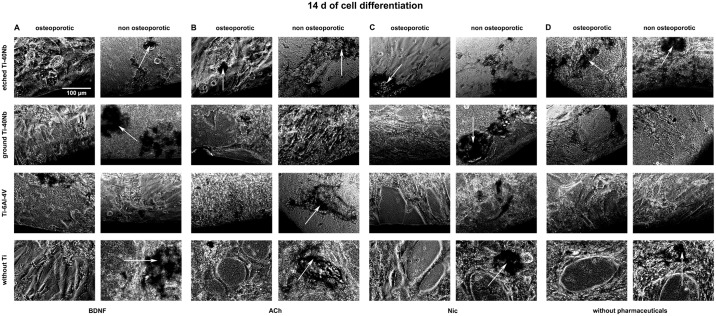
Live cell images of hMSCs after 14 days of differentiation in osteogenic medium *in vitro*. Shown are hMSCs of osteoporotic (left) and non osteoporotic (right) donors in presence of etched (1^st^ row) or ground Ti-40Nb (2^nd^ row), Ti-6Al-4V (3^rd^ row) or without Ti (4^th^ row) in presence of BDNF (A), ACh (B), Nic (C) or without pharmaceuticals serving as controls (D). White arrows indicate mineral. The images show cells of different donors as typical representative of 4 independent experiments. Black regions at the margin of pictures show Ti alloys. Scale bar shown in A applies to all photographs in this figure.

**Fig 6:**
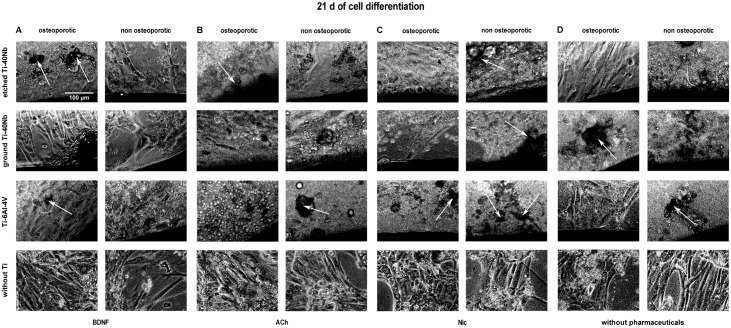
Live cell images of hMSCs after 21 days of differentiation in osteogenic medium *in vitro*. Shown are hMSCs of osteoporotic (left) and non osteoporotic (right) donors in presence of etched (1^st^ row) or ground Ti-40Nb (2^nd^ row), Ti-6Al-4V (3^rd^ row) or without Ti (4^th^ row) in presence of BDNF (A), ACh (B), Nic (C) or without pharmaceuticals serving as controls (D). White arrows indicate mineral. The images show cells of different donors as typical representative of 4 independent experiments. Black regions at the margin of pictures show Ti alloys. Scale bar shown in A applies to all photographs in this figure.

Live cell imaging of hMSCs showed that cells of osteoporotic and non osteoporotic donors orientated towards all tested Ti alloys and did not avoid contact to the material (Figs 1A–1D–6A–6D). Predominantly spindle-shaped morphology of hMSCs was seen during the entire evaluation period. Less often large, flat hMSCs were observed. Moreover, rapidly self-renewing cells (RS cells) were predominantly present in cultures of hMSCs of non osteoporotic donors. After 21 d of culture cell number (Fig 3) and mineralization (Fig 6) were visually increased in comparison to cell number and mineralization at time point 7 d (Figs 1 and 4). Images of cell number at time point 14 d are shown in Fig 2. Less mineral was present after 7 d (Fig 4) of osteogenic differentiation compared to time points 14 d (Fig 5) and 21 d (Fig 6). At time point 14 days more mineralization was observed in cell cultures of non osteoporotic donors compared to cells of osteoporotic donors (Fig 5). In contrast, after 21 d more mineral was seen in cell cultures of osteoporotic donors compared to non osteoporotic donors (Fig 6).

### 3.2 Immunofluorescence imaging

**Fig 7:**
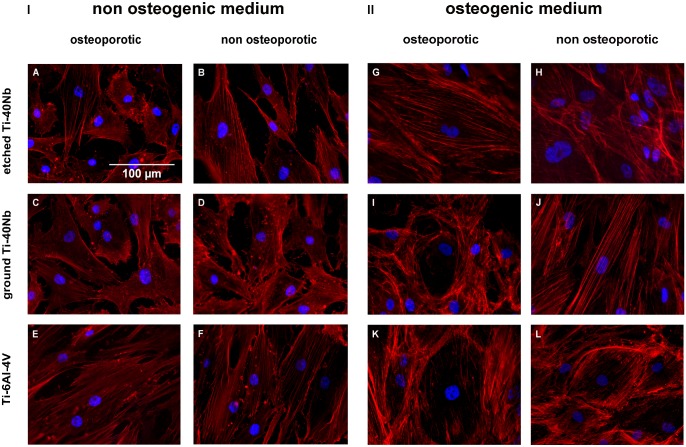
Immunofluorescence labeling of hMSCs incubated in non osteogenic and osteogenic medium. (I) hMSCs of osteoporotic (left) and non osteoporotic (right) donors after 1 d of incubation with etched (A and B), ground Ti-40Nb (C and D) or Ti-6Al-4V (E and F) in presence of non osteogenic medium without pharmaceuticals. (II) hMSCs of osteoporotic (left) and non osteoporotic (right) donors after 7 d of incubation with etched Ti-40Nb (G and H), ground Ti-40Nb (I and J) and Ti-6Al-4V (K and L) in presence of osteogenic medium without pharmaceuticals.

Immunofluorescence labeling of hMSCs showed that cells attached to all Ti alloys tested after 1 d of incubation in non osteogenic medium (I) as seen in Fig 7A–7F and after 7 d of incubation in osteogenic medium (II) shown in Fig 7G–7L. Whereas in non osteogenic medium cells attached to the material in a rather large, elongated and flat shape (Fig 7A–7F), they occasonally formed a round cytoskeleton in osteogenic medium. This was seen for cells of osteoporotic donors incubated with ground Ti-40Nb (Fig 7I) and cells of both donor pools incubated with Ti-6Al-4V (Fig 7K and 7L).

### 3.3 Determination of hMSCs numbers

**Fig 8:**
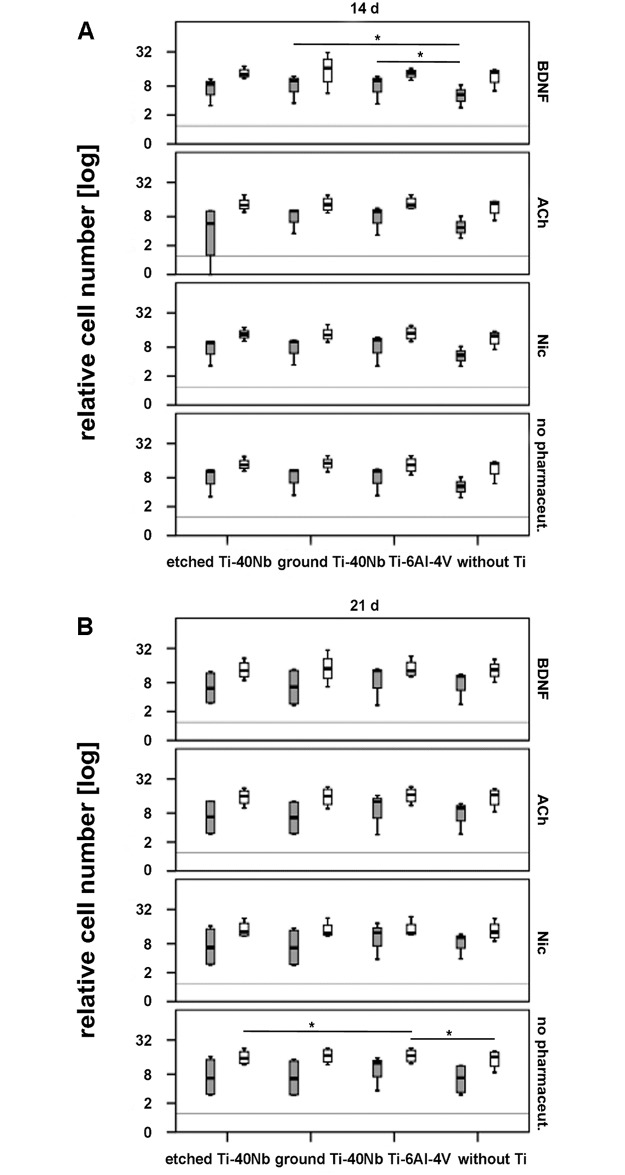
Relative cell number of hMSCs of osteoporotic (grey boxplots) and non osteoporotic (white boxplots) donors in presence of etched or ground Ti-40Nb, Ti-6Al-4V as well as without Ti with or without pharmaceuticals. Shown is the effect of Ti alloys on cell number after 14 d (A) and 21 d (B) of *in vitro* incubation. The grey line represents cells at time point 0 d without Ti and without pharmaceuticals. A value of p ≤ 0.05 was considered to be significant and is indicated with one asterisk.

An increase in relative cell number was detected generally for hMSCs of osteoporotic and non osteoporotic donors in presence of etched and ground Ti-40Nb as well as Ti-6Al-4V, with or without pharmaceuticals within 7–21 d (Fig 8 and supporting information S2 Fig).

A significant increase of relative cell number was measured for hMSCs of osteoporotic donors after 14 d of incubation with BDNF in presence of ground Ti-40Nb or Ti-6Al-4V compared to hMSCs that were incubated with BDNF but without Ti (Fig 8A).

After 21 d of cell culture Ti-6Al-4V without pharmaceuticals was significantly more stimulating than etched Ti-40Nb without pharmaceuticals on the proliferation of hMSCs of non osteoporotic donors. Moreover, number of hMSCs of non osteoporotic donors was increased in presence of Ti-6Al-4V compared to hMSCs of the same donor pool that were incubated without Ti and without pharmaceuticals (Fig 8B).

### 3.4 Analysis of cell differentiation based on ALP activity

**Fig 9:**
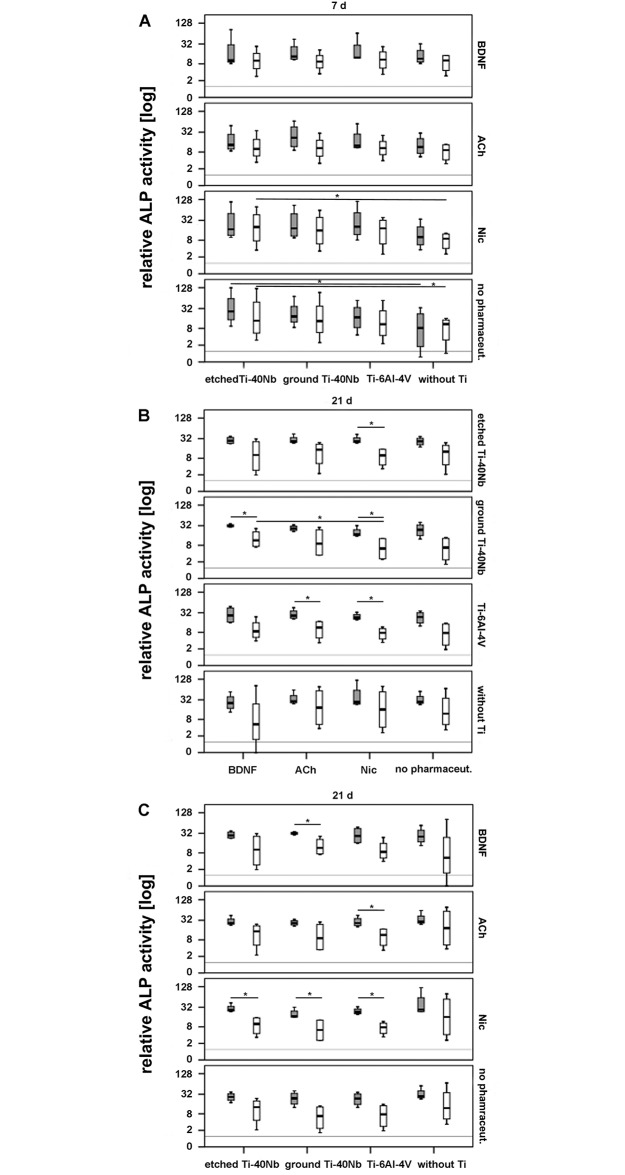
Relative ALP activity in hMSCs of osteoporotic (grey boxplots) and non osteoporotic donors (white boxplots) in presence of etched or ground Ti-40Nb, Ti-6Al-4V as well as without Ti with or without pharmaceuticals. Shown are the effect of Ti alloys on ALP activity after 7 d (A) and 21 d (B) as well as the effect of pharmaceuticals after 21 d of *in vitro* incubation (C). The grey line represents cells at time point 0 d without Ti and without pharmaceuticals. A value of p ≤ 0.05 was considered to be significant and is indicated with one asterisk.

After 7 days of incubation etched Ti-40Nb in absence of pharmaceuticals was the most stimulating titanium alloy on ALP activity of cells of osteoporotic and non osteoporotic donors. A significant increase occurred in comparison to cells that were incubated without Ti (Fig 9A). Moreover, a significant increase in relative ALP activity was detected in cells of non osteoporotic donors in presence of etched Ti-40Nb and Nic after 7 days of incubation when compared to cells that were incubated with Nic but without titanium (Fig 9A). Supporting information is shown in S3A Fig.

After 14 days of incubation no differences were detected between the different pharmaceuticals or titanium alloys in regard to relative ALP activity in cells of both donor pools (data shown in supporting information S3A and S3C Fig).

However, after 21 days in presence of etched Ti-40Nb, Nic was the most effective pharmaceutical on relative ALP activity of cells of osteoporotic donors compared to cells of non osteoporotic donors that were treated the same way (Fig 9B). In presence of ground Ti-40Nb, BDNF and Nic were the most effective pharmaceuticals on cell differentiation of osteoporotic donors, resulting in significantly higher ALP activity compared to cells of non osteoporotic donors. The effect of BDNF was even stronger than that of Nic when comparing the ALP activity of non osteoporotic donors (Fig 9B).

Nevertheless, Nic caused a significant increase in relative ALP activity in cells of osteoporotic donors compared to those of non osteoporotic donors, regardless of the titanium alloy added. This effect was shown for ACh only in presence of Ti-6Al-4V (Fig 9B).

Comparing the relative ALP activity of cells of both donor pools in absence of pharmaceuticals, none of the titanium alloys tested was significantly more effective than the other (Fig 9C).

## 4. Discussion

Cell imaging revealed that hMSCs of both donor pools attached to all Ti alloys tested. This aspect indicates that etching or grinding of the Ti-40Nb surface does not negatively affect cell adherence and is similar to the adherence of cells to Ti-6Al-4V.

However, immunofluorescence labeling showed that cell morphology was partially different between cells that were incubated in non osteogenic or osteogenic medium. After 7 d in presence of osteogenic medium and ground Ti-40Nb as well as Ti-6Al-4V, cells developed a roundish looking (nest-like) cytoskeleton. The nest-like cytoskeleton is considered to be characteristic of not fully differentiated osteoblasts [37]. According to Owen et al. (1990) ALP activity, as a marker for osteoblastic differentiation, should be the highest at this stage [38]. In our study cells with nest-like structure were detected after 7 d on ground Ti-40Nb and Ti-6Al-4V. However, 7 d results of the ALP assay showed that activity was the highest in presence of etched Ti-40Nb. This observation matches Rodriguez et al. (2004) who ascertained that hMSCs can reveal the characteristic cytoskeleton of differentiated cells but exhibit low ALP activity [39].

Generally, the number of hMSCs was rarely altered. After 14 d BDNF was the only pharmaceutical that caused a significant effect on the number of hMSCs of osteoporotic donors in presence of ground Ti-40Nb or Ti-6Al-4V when compared to cells that were incubated with BDNF but without Ti. Cells of non osteoporotic donors remained unaffected. However, after 7 d BDNF did not increase the number of hMSCs. This corresponds with the result of Ida-Yonemochi et al. (2017) who did not detect an increase in proliferation in MC3T3-E1 cells after 7 d [40]. In contrast, Cai et al. (2010) showed that osteoblast proliferation increased after 6 d in a co-culture system of BDNF-producing Schwann cells and osteoblasts [41]. We therefore hypothesize that BDNF shows time-dependent effects on the number of hMSCs in presence of ground Ti-40Nb or reference Ti.https://www.ncbi.nlm.nih.gov/pubmed/?term=Ida-Yonemochi%20H%5BAuthor%5D&cauthor=true&cauthor_uid=28072837 ACh and Nic did not significantly increase the number of hMSCs at any time point.

After 21 d of *in vitro* cultivation, hMSCs numbers of non osteoporotic donors were significantly increased in presence of Ti-6Al-4V and without pharmaceuticals when compared to hMSCs that were incubated with etched Ti-40Nb or without Ti.

Pharmaceuticals alone did not alter numbers of hMSCs. This might be reasoned by the concentration of pharmaceuticals used. We used an ACh concentration of 10^−4^ M, which neither significantly increased nor decreased hMSCs numbers. Another study analyzing proliferation of bone marrow-derived MSCs from rats, applied ACh concentrations ranging from 10^−5^–10^−9^ M. These concentrations did not affect proliferation either [42].

A dose-dependent effect was detected for Nic by Kim et al. (2012) who showed a significant increase of cell proliferation after 7 d of incubation using Nic concentrations of 1 or 2 mM [33]. A Nic concentration of 1 μM, as applied in our study, did not affect the proliferation of cells [33], which is in accordance with our results.

On the other hand, a concentration of 1 μM Nic significantly increased the differentiation of cells of non osteoporotic donors after 7 d in presence of etched Ti-40Nb, but also without Nic differentiation of hMSCs of both donor pools increased significantly after 7 d in presence of etched Ti-40Nb.

After 14 d of differentiation ALP activity was the same in both donor pools, irrespective of the titanium alloy or pharmaceutical tested.

In contrast to Sato et al. (2010) who showed that ACh decreased ALP activity in murine osteoblasts *in vitro* [29], we detected an increase in ALP activity of hMSCs of osteoporotic donors in presence of ACh and Ti-6Al-4V after 21 d.

Usually, ALP activity of osteoblasts decreases with progressing mineralization [38]. However, after 21 d ALP activity in hMSCs of osteoporotic donors was significantly higher when compared to hMSCs of non osteoporotic donors. In fact, ALP activity in hMSCs of non osteoporotic donors decreased significantly after 21 d of differentiation in presence of all titanium alloys and pharmaceuticals tested. This indicates that the peak of ALP activity was reached earlier in cells of non osteoporotic donors and mineralization had already started, which led to the decrease in ALP activity after 21 d. This is in accordance with live cell images, which showed a rather large amount of mineral after 14 d in cell cultures of non osteoporotic donors compared to those of osteoporotic donors. Furthermore, we detected RS cells in cultures of non osteoporotic donors. RS cells are a hMSC subtype which were shown to differentiate more extensively than larger mature hMSCs [43]. This is a further indicator that differentiation was already progressed in hMSCs of non osteoporotic donors. Moreover, our results suggest that low concentrations of BDNF, ACh or Nic stimulate ALP activity in hMSCs of osteoporotic donors after 21 d in presence of all titanium alloys. Supporting this statement is the aspect that ALP activity did not differ significantly between both donor pools after 21 d when incubated without pharmaceuticals in presence of each titanium alloy. Stimulation of osteoblast differentiation is aimed in osteoporotic patients since it is known that in osteoporosis the potential to differentiate into osteoblasts is declined and differentiation into adipocytes is increased [44].

Guo et al. (2016) found that BDNF knockdown can suppress marker expression of osteoblastic differentiation, indicating that BDNF might stimulate osteoblast differentiation [45]. We observed that BDNF significantly increased cell differentiation of non osteoporotic donors in presence of ground Ti-40Nb compared to the same cells that were incubated with ground Ti-40Nb but in presence of Nic. This result shows that BDNF is more stimulating than Nic on cell differentiation of non osteoporotic donors in presence of ground Ti-40Nb after 21 d.

However, together with etched Ti-40Nb or Ti-6Al-4V, Nic increased cell differentiation of osteoporotic donors as well.

Even though hMSCs numbers and differentiation were also increased after 21 d in presence of Ti-6Al-4V, the contained elements vanadium and aluminum are known to be toxic [4, 6, 46]. Particularly, vanadium can cause allergic reactions, which led to eczematous dermatitis and implant failure in one patient [47]. Moreover, Ti-6Al-4V possesses a relatively high elastic modulus (approx. 110 GPa) compared to that of human bone [11], which makes it rather inapplicable for orthopedic surgery, especially of osteoporotic bone. Ti-40Nb possesses a low elastic modulus of approximately 60–62 GPa [5], which comes closer to that of bone (cortical bone: 16–20 GPa, cancellous bone: 1–4 GPa) [4, 8, 9] when compared to Ti-6Al-4V. This indicates that etched or ground Ti-40Nb might be more suitable as implant materials than Ti-6Al-4V. Besides, Nb is a non toxic element [11].

Implant surfaces can determine cell behavior. The interaction between cells and material can regulate processes such as proliferation or differentiation of cells [11]. It was shown that rough titanium implant surfaces increased differentiation of hMSCs towards the osteoblastic lineage [48], which is aimed in regard to osseointegration of implants. Besides, chemical properties of implant material can influence cell behavior [11]. In our study, differentiation of hMSCs of non osteoporotic donors into osteoblasts was achieved after 7 d either in presence of etched Ti-40Nb together with Nic or in both donor pools without any pharmaceutical also in presence of etched Ti-40Nb. After 21 d all titanium alloys tested increased differentiation of hMSCs of osteoporotic donors in presence of each pharmaceutical.

To summarize, hMSCs numbers of osteoporotic donors increased after 14 d in presence of ground Ti-40Nb or Ti-6Al-4V, both accompanied by BDNF. hMSCs numbers of non osteoporotic donors increased significantly after 21 d in presence of Ti-6Al-4V only. However, Ti-6Al-4V is not preferred because of the toxic effects of aluminum and vanadium.

In regard to osteoblast differentiation ground Ti-40Nb together with BDNF was effective in both donor pools.

Considering that hMSCs numbers and differentiation were both significantly increased in presence of ground Ti-40Nb and BDNF, we conclude that this alloy might be the most suitable candidate for *in vivo* applications.

## Supporting information

S1 Fig Determination of the appropriate concentration of BDNF (A), ACh (B) and Nic (C) for use in experiments. Shown are the different pharmaceutical concentrations compared to cells treated without pharmaceuticals (control). Values above bars indicate ALP concentrations in percentage compared to the control.
(TIF)

S2 Fig Relative cell number of hMSCs of osteoporotic (grey boxplots) and non osteoporotic (white boxplots) donors in presence of etched or ground Ti-40Nb, Ti-6Al-4V as well as without Ti with or without pharmaceuticals. Shown are the effects of Ti alloys and pharmaceuticals on cell number after 7 d (A and B) and 14 d (C) and 21 d (D) of *in vitro* incubation. The grey line represents cells at time point 0 d without Ti and without pharmaceuticals.
(TIF)

S3 Fig Relative ALP activity in hMSCs of osteoporotic (grey boxplots) and non osteoporotic donors (white boxplots) in presence of etched or ground Ti-40Nb, Ti-6Al-4V as well as without Ti with or without pharmaceuticals. Shown are the effects of pharmaceuticals and Ti alloys on ALP activity after 7 d (A) as well as after 14 d (B and C) of *in vitro* incubation. The grey line represents cells at time point 0 d without Ti and without pharmaceuticals.
(TIF)
