# Supplementary figures and images for: Effects of new beta-type Ti-40Nb implant materials, brain-derived neurotrophic factor, acetylcholine and nicotine on human mesenchymal stem cells of osteoporotic and non osteoporotic donors
Source: PLoS One. 2018 Feb 28;13(2):e0193468. doi: 10.1371/journal.pone.0193468 (PMC5873971; doi:10.1371/journal.pone.0193468)

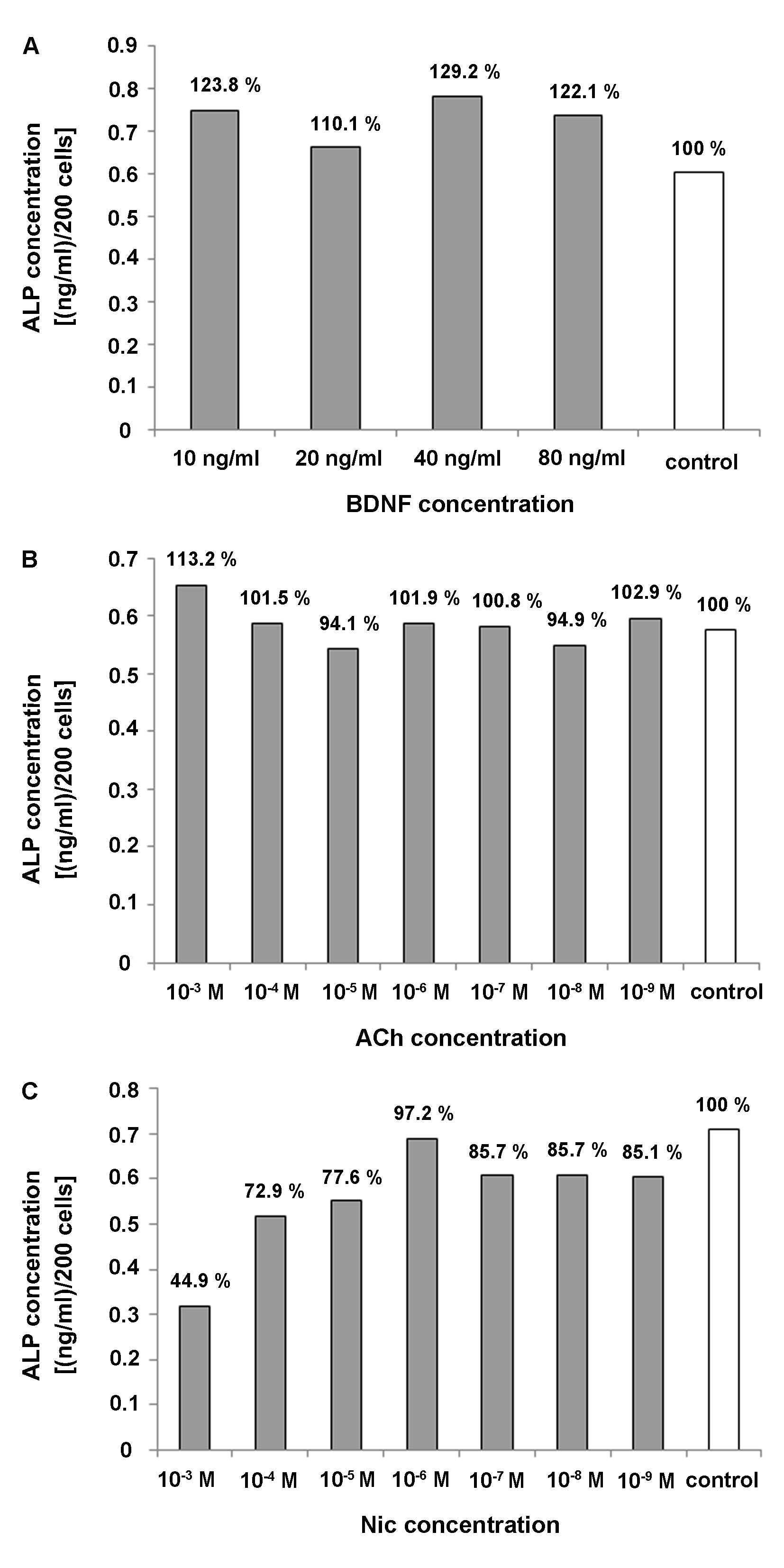

Supplement: S1 Fig — Shown are the different pharmaceutical concentrations compared to cells treated without pharmaceuticals (control). Values above bars indicate ALP concentrations in percentage compared to the control. (TIF) [file pone.0193468.s001.tif]

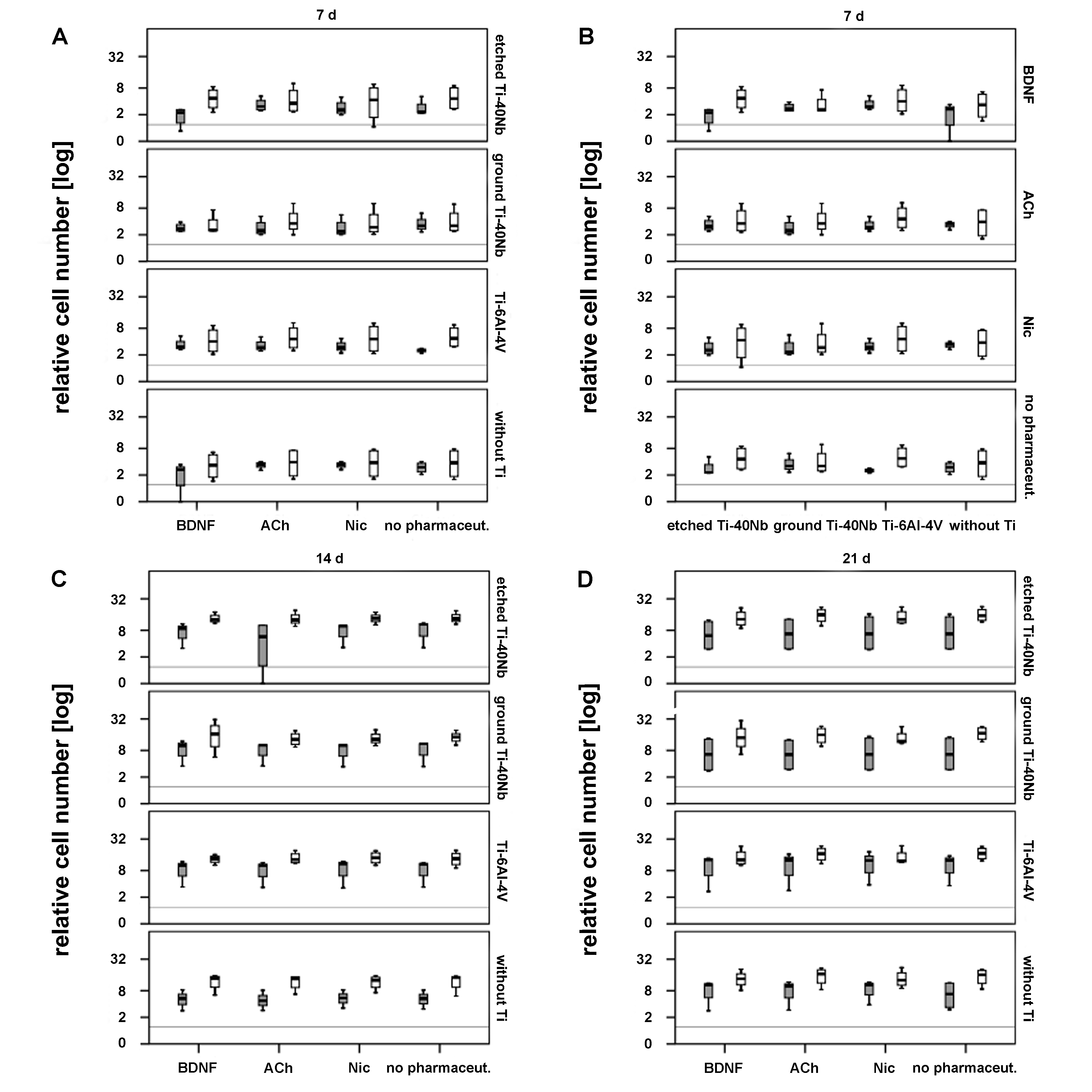

Supplement: S2 Fig — Shown are the effects of Ti alloys and pharmaceuticals on cell number after 7 d (A and B) and 14 d (C) and 21 d (D) of in vitro incubation. The grey line represents cells at time point 0 d without Ti and without pharmaceuticals. (TIF) [file pone.0193468.s002.tif]

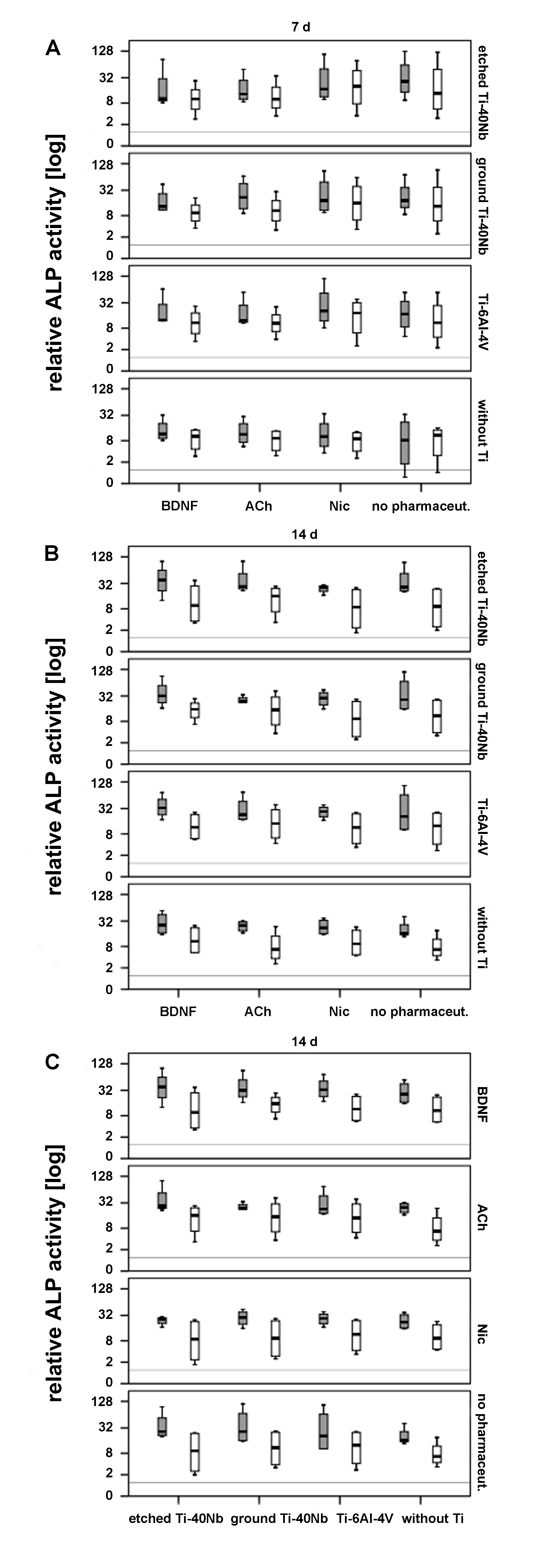

Supplement: S3 Fig — Shown are the effects of pharmaceuticals and Ti alloys on ALP activity after 7 d (A) as well as after 14 d (B and C) of in vitro incubation. The grey line represents cells at time point 0 d without Ti and without pharmaceuticals. (TIF) [file pone.0193468.s003.tif]
